# Sildenafil Effect on Nitric Oxide Secretion by Normal Human Endometrial Epithelial Cells Cultured *In vitro*

**Published:** 2011-12-22

**Authors:** Mozafar Khazaei, Shiva Roshankhah, Rostam Ghorbani, Farzaneh Chobsaz

**Affiliations:** Fertility and Infertility Research Center, Kermanshah University of Medical Sciences, Kermanshah, Iran

**Keywords:** Epithelial Cells, Sildenafil, Endometrium, Nitric Oxide

## Abstract

**Background:**

Sildenafil is a selective inhibitor of cyclic-guanosine monphosphat-specific phosphodiesterase type 5. It increases intracellular nitric oxide (NO) production in some cells. There are reports on its positive effect on uterine circulation, endometrial thickness, and infertility improvement. Endometrial epithelial cells (EEC) play an important role in embryo attachment and implantation. The present work investigates the effect of sildenafil on human EEC and their NO secretion *in vitro*.

**Materials and Methods:**

In this experimental *in vitro* study, endometrial biopsies (n=10) were washed in a phosphate buffered solution (PBS) and digested with collagenase I (2 mg/ml in DMEM/ F12 medium) at 37°C for 90 minutes. Epithelial glands were collected by sequential filtration through nylon meshes (70 and 40 μm pores), respectively. Epithelial glands were then treated with trypsin to obtain individual cells. The cells were counted and divided into four groups: control and 1, 10, and 20 μM sildenafil concentrations. Cells were cultured for 15 days at 37ºC and 5% CO2; the media were changed every 3 days, and their supernatants were collected for the NO assay. NO was measured by standard Greiss methods. Data were analyzed by one way ANOVA.

**Results:**

There was no significant difference between groups in cell count and NO secretion, but the level of NO increased slightly in the experimental groups. The 10 μM dose showed the highest cell count. EEC morphology changed into long spindle cells in the case groups.

**Conclusion:**

Sildenafil (1, 10, and 20 μM) showed a mild proliferative effect on human EEC numbers, but no significant change was seen in NO production.

## Introduction

The endometrium is an important part of the female reproductive tract and plays a pivotal role in uterine pathophysiology. Human endometrium is a unique and dynamic tissue which has an intensive period of proliferation, growth, angiogenesis, remodeling, and destruction (1, 2). The endometrium plays a pivotal role in the implantation process and one of its measurable characteristics is its epithelial responsiveness. The epithelial layer of endometrium is the first maternal part that accepts an implanting blastocyst. Endometrial epithelial and stromal cells have specific morphological and functional properties (3, 4).

Sildenafil is a member of the 5-phosphodisterase (5PDE) inhibitor, which hydrolyzes destructive enzymes of cyclic guanosine monophosphate (cGMP) and increases the intracellular level of both cGMP and nitric oxide (NO) (5). Sildenafil is also responsible for the degradation of cGMP in the corpus cavernosum. The molecular structure of sildenafil is similar to cGMP and acts as a competitive binding agent of PDE5 (6).

NO is a small, multi-faced molecule with a regulatory role in many areas of biology. It diffuses the cell membrane freely and controls the physiologic and pathologic function of the cardiovascular, immune, and nervous systems (7, 8).

The biological role of NO was first detected in the macrophages and neutrophils of rodents(9). It is released from different cells including smooth muscle, neurons, platelets, hepatocytes, macrophages, fibroblasts, mesengeal and epithelial cells. NO regulates smooth muscle contraction, platelet aggregation and attachment, cell growth, apoptosis, and the immune responses of inflammation (10).

The role of NO in uterine biology and pathophysiology is defined by the regulation and spontaneous contraction of the myometrium during pregnancy. Uterine circulation-induced NO synthetase enzyme (NOS) is found in vessel walls, neurons, glandular epithelium, endometrial stromal cells, myometrial stromal cells, and mast cells (11).

Studies show that vaginal sildenafil improves sexual response and endometrial receptivity, and it can cure the sexual function of menopause women (12). A study demonstrated that NO and progesterone show synergistically induced apoptosis in endometrial epithelial cells (EEC) (13). Also, the effect of sildenafil on cultured human coronary endothelial cells have been studied, in which 1, 10, and 20 μM of sildenafil showed both growth and angiogenic effect on these cells (14).

After looking at other literature, there are no reports on the effect of sildenafil on EEC. The aim of this study, therefore, is to investigate the effect of sildenafil on the numbers and morphology of EEC and their NO secretion *in vitro*.

## Materials and Methods

### Sample collection

In this experimental *in vitro* study, endometrial biopsies (n=10) were taken from women of reproductive age (25-40 years old) who underwent surgery for either benign myoma or diagnostic laparoscopy. Each sample was divided into two parts, one for pathologic diagnosis and the other for cell culture. Endometrial malignancies (polyps, hyperplasia, and cancer) and patients with hormone therapy were excluded. Endometrial samples were in the proliferative phase. The Ethics Committee of Kermanshah University of Medical Sciences accepted the work on human tissue in this study and all patients signed informed consents.

### Culture methods

Endometrial biopsies were washed in PBS that contained a 2% antibiotic - antimycotic solution (Sigma, Germany). The biopsies were chopped in a 2 mg/ml collagenase I solution (Sigma, Germany) in DMEM/F12 media (Gibco, Denmark) and incubated at 37°C for 90 minutes. Cell suspensions were passed through 70 and 40 μm filter mesh (cell strainer; Becton Dickenson Company, USA). The 40 μm filter mesh was washed back to collect endometrial glands (15). Endometrial epithelial glands were dissociated into individual EEC by trypsin enzyme (0.025%). Trypan blue staining was used for cell viability and DAKO standard methods were done for cytokeratin as an epithelial cell marker (16, 17).

The cells were divided into four groups. The control group received DMEM/F12 media that contained a 1% antibiotic–antimycotic solution supplemented by 5% fetal bovine serum and 2 ìM L-glutamine. Experimental groups received the same media and either 1, 10 or 20 ìM sildenafil doses. These doses were selected based on pervious work (14). The culture period was 15 days and the culture media were changed every 3 days. On the first and last day of the culture, cells were photographed. During the culture period cell growth and morphological changes were studied. At the end of the study, the cells were harvested by trypsin-EDTA (0.25%). Cell numbers and viability were detected by trypan blue staining.

### Nitric oxide assay

With a 6-10 second half-life, NO is very unstable and rapidly converts to nitrite in media that contains oxygen. NO concentration in the supernatant was determined with the Greiss method (18). The Greiss reagent is made up of a 1% solution of sulfanilamide in 5% phosphoric acid and 0.1% naphthylethylenediamine dihycrochloride in distilled water.

Epithelial cell supernatants were collected each time the media was changed and kept at -20° C. The protein and phenol red of the supernatant were deleted using Zinc sulfate (6 mg/400 μliter) (19).

Sodium nitrite (0.1 M) was used for the standard curve, and increasing concentrations of sodium nitrite (5, 10, 25, 50, 75, and 100 μM) were prepared. The Greiss solution was added to all microplates containing sodium nitrite and supernatant and was read by an ELISA reader (stat fax100. USA) in 540 nm and 630 nm filters (20).

### Statistical analysis

Data were analyzed by one way analysis variance and post hoc Tukey test. P<0.05 was considered significant.

## Results

**Fig 1:**
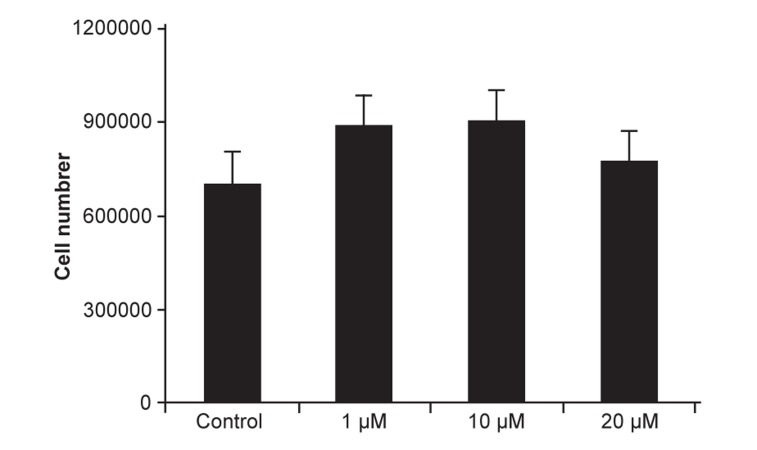
EEC means number in control and experimental groups.

Cell confluency was almost the same between the control and case groups, with no significant difference in final cell numbers (p=0.526). The 10 μM dose showed the highest cell numbers (Fig 1).

**Fig 2:**
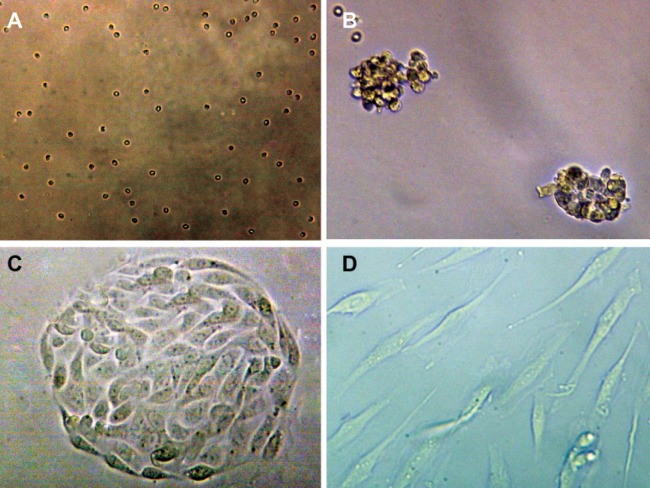
EEC (A) ×200 and epithelial glands (B) ×400 at beginning of the culture. Epithelial cell island (C) at the end of first week. Spindle EEC (D) during second week ×400.

The cell viability assay with trypan blue staining showed that the cells were alive at the end of the study and sildenafil did not have a toxic effect on them. The EECs were spheroid after collagenase digestion of the endometrial tissue (Fig 2A, B). The cells attached to the culture dish during the first day of the study. On the third day, cellular islands with polygonal EEC were seen (Fig 2C).

**Fig 3:**
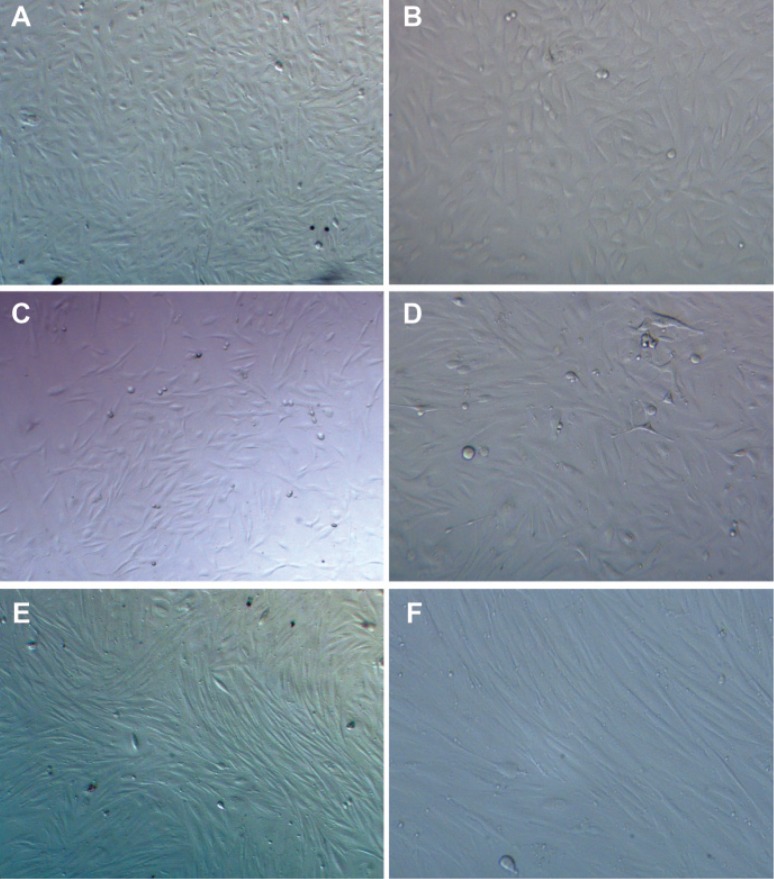
EEC at the end of the study. Control group: (A) ×200. (B) ×400. 1 μM group: (C) × 200, (D) ×400, 10 μM, (E) ×200, (F) ×400.

At the end of the first week, the EEC had a polygonal to spindle shape, and during the second week, they became long spindle shaped (Fig 2D). The longest spindle cells were seen in the 10 μM cultures. EECs showed a homogenous population in the culture dish (Fig 3) and at the end of the second week some EECs had granular and vacuolar cytoplasms with detachment from the culture dish, especially in the 20 μM group.

### Nitric Oxide changes

**Fig 4:**
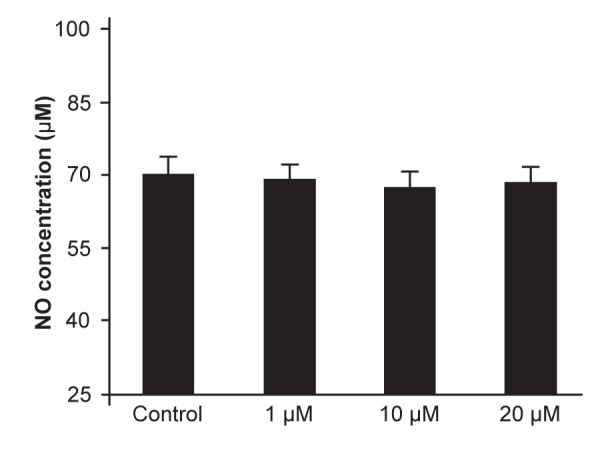
NO concentration (μM) mean in control and experimental groups.

The means of NO were 70.17 in the control group, 69.55 in the 1 ìM, 66.53 for 10 ìM, and 68.52 for 20 ìM doses of sildenafil. There was no significant difference in NO secretion between the control and case groups (p=0.761, Fig 4), and between different days of the study period.

## Discussion

To our knowledge, the present study is the first report on the effect of sildenafil on human EEC in an *in vitro* culture. EECs showed a homogenous population in the culture dish with no significant difference in their number between groups. However, 10 μM concentrations had the highest mean, which has shown a proliferative, but not hyperplastic, effect of this agent on EEC. This finding is in partial agreement with work done on the sildenafil effect on human coronary endothelial cells (14) and in contrast to work that indicates an antiproliferative effect of sildenafil on human endothelial cells (21).

Some reports introduce sildenafil for the improvement of endometrial thickness and receptivity (22, 23). We did not find any sildenafil side effects on EEC proliferation and their NO secretion. It should examine *in vitro* effect of sildenafil on other endometrial cells. In the future, our team will investigate the effect of sildenafil on human endometrial explants in a three-dimensional culture system.

One of the aims of the present work was to measure NO secretion by EEC using Greiss methods.

In the present work, sildenafil did not change NO secretion. NO is an important regulator of the biology and physiology of the reproductive system. The complexity of its biological effects is a consequence of its numerous potential interactions with other molecules such as reactive oxygen species (ROS), metal ions, and proteins (24)

The effects of NO are modulated by both direct and indirect interactions that can be dose-dependant and cell-type specific. NO can induce apoptosis in some cell types and inhibit apoptosis in others. Low NO concentration can inhibit apoptosis, but a higher concentration of NO may be toxic and can induce cell death, if not by apoptosis then by necrosis (24). In this study, the 1 and 10 μM doses of NO are correlated with EEC proliferation, but the 20 μM dose does not. Induction of apoptosis by NO depends, in part, on cell types in different organ systems.

More studies have to be performed to determine the exact mechanisms of sildenafil on EEC.

## Conclusion

Sildenafil did not show inhibitory or excitatory effects on NO secretion by EEC and in lower doses, it exerted a proliferative effect.
